# Acute Limb Ischaemia during ECMO Support: A 6-Year Experience

**DOI:** 10.3390/life13020485

**Published:** 2023-02-10

**Authors:** Ihor Krasivskyi, Clara Großmann, Marit Dechow, Ilija Djordjevic, Borko Ivanov, Stephen Gerfer, Walid Bennour, Elmar Kuhn, Anton Sabashnikov, Parwis Baradaran Rahmanian, Navid Mader, Kaveh Eghbalzadeh, Thorsten Wahlers

**Affiliations:** 1 Department of Cardiothoracic Surgery, University Hospital Cologne, 50937 Cologne, Germany; 2 Department of Cardiothoracic Surgery, Heart Centre, Helios Hospital Siegburg, 53721 Siegburg, Germany

**Keywords:** ECMO, limb ischaemia, distal limb perfusion

## Abstract

The use of veno-arterial extracorporeal membrane oxygenation (VA-ECMO) for cardiogenic shock is rising. Acute limb ischaemia remains one of the main complications after ECMO initiation. We analysed 104 patients from our databank from January 2015 to December 2021 who were supported with mobile ECMO therapy. We aimed to identify the impact of acute limb ischaemia on short-term outcomes in patients placed on ECMO in our institution. The main indication for ECMO therapy was left ventricular (LV) failure with cardiogenic shock (57.7%). Diameters of arterial cannulas (*p* = 0.365) showed no significant differences between both groups. Furthermore, concomitant intra-aortic balloon pump (IABP, *p* = 0.589) and Impella (*p* = 0.385) implantation did not differ significantly between both groups. Distal leg perfusion was established in approximately 70% of patients in two groups with no statistically significant difference (*p* = 0.960). Acute limb ischaemia occurred in 18.3% of cases (n = 19). In-hospital mortality was not significantly different (*p* = 0.799) in both groups. However, the bleeding rate was significantly higher (*p* = 0.005) in the limb ischaemia group compared to the no-limb ischaemia group. Therefore, early diagnosis and prevention of acute limb ischaemia might decrease haemorrhage complications in patients during ECMO therapy.

## 1. Introduction

Cardiovascular diseases are one of the most important causes of morbidity and mortality worldwide [1]. Atherosclerosis is one of the pathogenic causes of cardiovascular disease progression, which leads to a reduced arterial perfusion of vital organs [1,2]. Despite the improvement in therapeutic options, the incidence of atherosclerosis and relating complications remains high [2]. Therefore, prevention of cardiovascular diseases continues to be the main goal for both clinical physicians and researchers [1,2].

Veno-arterial extracorporeal membrane oxygenation (VA-ECMO) is increasingly used for the aforementioned patients when they present with cardiogenic shock or with cardiac arrest [3]. There are two different implantation strategies: a central ECMO and a peripheral ECMO. For a central ECMO, the right atrium and the ascending aorta are cannulated directly, and the thorax must be left open. Central ECMO provides a better perfusion of the upper extremities and brain caring a higher risk for infection and bleeding [4]. It is often used for patients with a post-cardiotomy shock and only rarely for emergencies. Due to the cannulation strategy, the peripheral ECMO is usually the method of choice in these emergent situations; it does not require an opening of the chest and can be established percutaneously. The most commonly used vessels for implanting a peripheral ECMO are the femoral artery and vein [5,6]. Peripheral ECMO can, however, also cause various complications such as bleeding, infection and limb ischaemia [7].

Acute limb ischaemia is a feared complication after peripheral VA-ECMO initiation and can have a devastating impact on patients’ outcomes and the survival rate [8]. It can result in inadequate blood supply to the ipsilateral limb, which can lead to the amputation or even, in the worst case, to the patient’s death [5,9]. The incidence of limb ischaemia is approximately 17% in the current literature [10]. It is often caused by various factors, such as the patients’ comorbidities, haemodynamic factors with low blood flow or the size of the cannulas [9]. Consequently, early diagnosis, prevention and treatment of this serious complication are the crucial goals for physicians in intensive care units [11,12]. The initiation of antegrade distal limb perfusion is one of the most important aims for preventing acute limb ischaemia in patients who are placed on VA-ECMO [13]. However, there is a lack of exact guidelines for the right timing and indication of the distal limb cannulation in the current literature [14,15,16].

We aimed to identify the impact of acute limb ischaemia on short-term outcomes in patients placed on ECMO in our institution. Therefore, we have analysed data from our mobile ECMO Retrieval program from 2015 to 2021 years.

## 2. Materials and Methods

This study was designed as a retrospective single-centre study. We included patients who underwent mobile ECMO therapy (Cardiohelp, Maquet, Rastatt, Germany) from January 2015 to December 2021. The sample size equals 111 patients. However, we excluded seven patients due to a lack of data. Therefore, 104 patients were divided into 2 groups: patients who suffered acute limb ischaemia (n = 19) and patients without ischaemia problems (n = 85). A certain part of the data has been previously described [17].

### 2.1. ECMO-Center Protocol

Our mobile ECMO program and protocol have already been described elsewhere [17]. All implantations were performed on-site in tertiary hospitals. Contact was usually initiated by the referring clinic. The patients were evaluated on site for ECMO therapy by a clinical examination and transthoracic echocardiography (TTE). The ECMO therapy was implemented according to the Extracorporeal Life Support Organization (ELSO) recommendations [18].

ECMO cannulation was usually performed per punctionem using the femoral artery and vein. Some of the cannulations were conducted without any imaging due to the lack of an ultrasound machine in the referring hospitals. In every case, there was an attempt to establish a distal limb perfusion. If the initial attempt failed, there was a repeated attempt at our centre.

Intravenous, unfractionated heparin with an activated clotting time (ACT) between 180 and 220 s and an activated partial thromboplastin time (aPTT) between 60 and 80 s was used to prevent potential thromboembolic events. ECMO therapy was performed with a mobile Cardiohelp (Maquet, Rastatt, Germany) machine in all cases.

Echocardiography, chest X-ray and the assessment of laboratory parameters were conducted daily to assess the patient’s haemodynamic situation. Transoesophageal echocardiography was used to evaluate the left and right ventricular function and to analyse the possibly weaning capacity. We increased the pump flow from 100–200 mL/hour up to 1.5 L/min. The lower limit of mean arterial pressure (MAP) was set to ≥60 mm/Hg. Lactate level and urine output were measured hourly.

We have established different protocols in our centre to diagnose limb ischaemia early. The legs of patients with ECMO were clinically examined every hour. In addition, leg circumference was measured every six hours, and Doppler sonographic evaluation was also performed every six hours. Laboratory parameters such as CK and CK-MB were determined daily as enzyme concentration, whereas lactate was monitored hourly.

Weaning was initialised after the patient reached haemodynamic stability. All patients who were assumed to be suitable for weaning underwent surgical explantation of the ECMO cannulas.

### 2.2. Data Collection

Data were obtained from our institutional database. We analysed:

- patients’ demographic characteristics (age (years), sex, body mass index (BMI, kg/m^2^));
- status before ECMO support (myocardial infarction, stroke, chronic obstructive lung disease (COPD), smoking status, chronic kidney disease (CKD), dialysis, diabetes, arterial hypertension, hyperlipidaemia and peripheral vascular disease (PVD));
- laboratory parameters (creatine kinase (CK), creatine kinase MB (CK-MB), lactate and creatinine);
- early outcome data (stroke, thromboembolic events, hepatic failure, gastrointestinal bleeding, renal failure and acute respiratory distress syndrome (ARDS)).

### 2.3. Outcome Analysis

We considered bleeding rate and in-hospital mortality to be primary outcomes. However, the secondary outcomes were dialysis, wound infection, septic shock and length of intensive care unit (ICU) stay.

### 2.4. Ethics

We performed this study according to the Declaration of Helsinki (as revised in 2013). Based on the decision of the Ethics Committee of the Medical Faculty of the University of Cologne, we do not need any ethical approval statements. According to German law, there is no need for additional ethical approval for purely retrospective clinical studies.

### 2.5. Statistical Methods

We performed statistical analysis using the Statistical Package for Social Sciences, version 28.1 (SPSS Inc., Chicago, IL, USA). Firstly, variables were checked for the sample distribution using the Kolmogorov–Smirnov Test. The Student’s *t*-Test was used for continuous variables which are normally distributed, whereas a Mann–Whitney U test was used for those which are not normally distributed. Categorical variables were analysed with a Chi-square test. All continuous variables with a normal distribution are presented as mean ± standard deviation (SD). Continuous variables with a skewed sample distribution were presented as median and min./max. range. Categorical variables are presented in percentages. We considered the *p*-value of Fisher’s exact test when the minimum expected count of cells was <5. We created both univariate and multivariate regressions in order to analyse combined symptoms. We considered a 95% CI and a *p*-value < 0.05 to be significant.

## 3. Results

**Figure 1:**
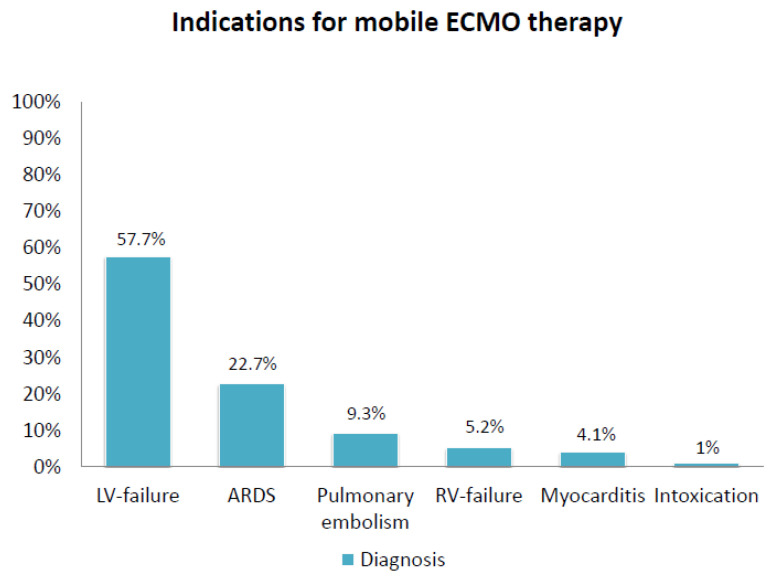
Indications for mobile ECMO-therapy.

All relevant reasons for ECMO initiation are shown in Figure 1. The main indication was left ventricular (LV) failure with cardiogenic shock (57.7%). ARDS (22.7%), pulmonary embolism (9.3%) and right ventricular (RV) failure (5.2%) were other indications for ERMO implantation. Only four patients (4.1%) suffered from acute myocarditis before ECMO therapy.

LV-failure: left ventricular failure; ARDS: acute respiratory distress syndrome; RV-failure: right ventricular failure.

### 3.1. Baseline Data

Baseline data of the two groups (limb ischaemia (n = 19) and no limb ischaemia (n = 85)) are presented in Table 1. In terms of demographic data and relevant comorbidities, the groups were nearly equal and showed no significant differences. The mean age in our sample was 56 years in the limb ischaemia group and 53 years in the no limb ischaemia group (*p* = 0.399). There were slightly more than 40% females in the limb ischaemia group, whereas in the no limb ischaemia group, approximately 30% were females (*p* = 0.333). Patients in both groups were, on average, overweight. The mean BMI in the limb ischaemia group was 29.8 kg/m^2^, whereas in the no limb ischaemia group, the mean BMI amounted to 28.4 kg/m^2^ (*p* = 0.543). Patients who suffered limb ischaemia also smoked more often in comparison to the no limb ischaemia group, 42.1% and 32.1%, respectively. However, despite the 10% difference, it was not statistically significant (*p* = 0.408). Peripheral arterial disease was not significantly higher (*p* = 0.458) in patients with limb ischaemia (5.3%) compared to patients without limb ischaemia (2.4%).

### 3.2. ECMO-Initiation Data

Table 2 shows all relevant ECMO-initiation data. Concomitant intra-aortic balloon pump (IABP, *p* = 0.589) and Impella (*p* = 0.385) implantation did not differ significantly between both groups. Furthermore, diameters of arterial cannulas (*p* = 0.365) showed no significant differences between two groups. Moreover, DPC was not performed significantly more often (*p* = 0.976) in patients without limb ischaemia compared to patients who suffered limb ischaemia. Vasopressor therapy showed no significant difference for three days between two groups (*p* = 0.668, *p* = 0.663, *p* = 706). The number of patients requiring vasopressor therapy decreased as time progressed.

### 3.3. Labaratory Data

The laboratory parameters at admission, 24 h, 48 h, 72 h and 96 h after ECMO implantation are shown in Table 3. We measured and compared CK, CK-MB, lactate and creatinine levels for both limb ischaemia and no limb ischaemia groups. According to our findings, after 96 h, CK (*p* = 0.002) and CK-MB (*p* = 0.002) levels were significantly higher in patients with limb ischaemia compared to patients without limb ischaemia. In contrast, lactate did not differ significantly (*p* = 0.693) after 96 h between both groups. Likewise, further data did not differ significantly between the groups.

### 3.4. Primary and Secondary Endpoints

Acute limb ischaemia occurred in 18.3% of cases (n = 19). Approximately 53% of patients with limb ischaemia had to undergo surgical embolectomy, and three patients (15.7%) required surgical fasciotomy. This was significantly more often than in the group without limb ischaemia. The bleeding rate was significantly higher (*p* = 0.005) in the limb ischaemia group compared to no limb ischaemia and three patients (15.7%) needed surgery due to the consistent bleeding (Table 4). In contrast, thromboembolic events did not differ significantly (*p* = 0.371) between two groups. Wound infection rate was comparable with 5.3% in limb ischaemia group and 7.1% in no limb ischaemia group (*p* = 0.769). Dialysis rate was quite similar (47.4% vs. 35.7%, *p* = 0.344) between both groups. Further relevant secondary outcomes did not differ significantly between two groups. Likewise, in-hospital mortality was not significantly higher (*p* = 0.799) in patients with limb ischaemia compared to patients without limb ischaemia.

## 4. Discussion

In our study, we looked at the impact of acute limb ischaemia on short-term outcomes of patients with veno-arterial ECMO therapy. Although the bleeding rate was significantly higher (*p* = 0.005) in the limb ischaemia group compared to the no limb ischaemia group, the mortality rate was not significantly different (*p* = 0.799) in both groups.

As already described, VA-ECMO is increasingly used as a rescue tool in emergency situations [8]. Previous researchers specified that limb ischaemia after VA-ECMO initiation was approximately 20% [19,20]. In our study, it was measured at 18.3%, which is comparable with findings of previous articles [21,22]. Moreover, there was no significant difference of in-hospital mortality (*p* = 0.799) between patients with limb and without limb ischaemia. This is contrary to other trials that have described limb ischaemia as an independent predictor of mortality [23,24].

Early diagnosis and treatment of acute limb ischaemia has been described in various papers independently of ECMO treatment [25,26]. The clinical diagnosis of limb ischaemia has been described by Pratt with the six “Ps”: pain, poikilothermic, paleness, pulselessness, paraesthesia and paralysis [27]. Therefore, clinical examination is crucial to detect limb ischaemia in a timely manner. As the patients are almost always intubated and sedated, paraesthesia, paralysis and, to a certain extent, pain cannot be detected easily. Thus, special attention must be paid to the temperature and colour of the leg and the pulse status. In our protocol, the legs were examined every hour, and circumference measurement and Doppler sonography were performed every six hours. In addition, we measured laboratory parameters such as lactate levels on an hourly basis. Therefore, we tried to identify an ischaemic leg as early as possible and initiate treatment. In our study, there was no significant difference in in-hospital mortality between patients with and without limb ischaemia (*p* = 0.799). This could potentially be due to an early diagnosis.

Moreover, there are different treatment options for limb ischaemia. Limb ischaemia due to an ECMO cannula can be reversed by removing the cannula. However, in most cases, this is not directly possible, as the patient’s life depends on ECMO. However, other ECMO cannulation sites or even a different aide such as an Impella can be evaluated in order to remove the cannula and reverse ischaemia [28]. In addition, vascular surgeons should be referred to promptly in order to evaluate the severeness of the ischaemia and the likelihood of further complications [29]. They can then decide if therapeutic intervention is necessary. In our study, 52.6% of patients with limb ischaemia required vascular surgery with embolectomy (*p* ≤ 0.001). In addition, three patients (15.7%) also required fasciotomy due to a compartment syndrome (*p* = 0.005). Tanaka et al. reported similar findings, with 20% of patients requiring surgical intervention and 12% requiring fasciotomy [11]. In the study by Yau et al., three patients even needed an amputation [30]. Therefore, an earlier diagnosis might help to reduce this number and save patients from these severe vascular complications.

The initiation of distal perfusion cannula (DPC) in patients on VA-ECMO support has been controversial [26,31,32,33]. Several authors defined the absence of a DPC as an independent risk factor for critical limb ischaemia [34]. Moreover, Spurlock et al. mentioned that distal limb cannulation should be performed in the first 6 h after VA-ECMO implantation to prevent potential ischaemic limb injury [35]. In addition, further studies showed that the use of DPC was associated with lower mortality after VA-ECMO implantation [36]. Furthermore, Russo et al. mentioned that the use of DPC could prevent limb ischaemia and oedema in patients on VA-ECMO [37]. In contrast, several studies showed that the use of DPC was not associated with improved outcomes after VA-ECMO initiation [32,38]. Moreover, the authors hypothesised that an additional arterial puncture might be associated with potential vascular complications [38]. Obese patients or patients with vascular calcification are thereby most commonly at risk [39]. Jang et al. described the use of the fluoroscopy-guided insertion of the distal perfusion cannula as a method to reduce the complication rate [40]. In our study, the rate of distal limb perfusion was not statistically different between the two groups (*p* = 0.960) and was approximately 70% in both. The reason for not establishing a DPC was most often difficulties in the setup of the distal perfusion cannula.

Previous studies have described peripheral arterial disease as a predictor of vascular complications [31]. Hasegawa et al. even presented an extreme case in which ECMO cannulation could not be established due to obstruction and stenosis of the femoral artery [28]. In our study, peripheral arterial disease did not differ significantly between both groups (*p* = 0.458). A limiting aspect is that this describes only the pre-existing PAD that had been recorded at admission of the patient. Because this diagnosis is established primarily in clinical settings, the number of unreported cases is probably significantly higher. Therefore, patients with PAD should be evaluated very carefully at cannulation, and an open technique should be favoured [18].

Another factor to consider is the use of vasopressors. A high proportion of ECMO patients require therapy with vasopressors. The study by Newbury et al. looked at the effect of vasopressor use on acute limb ischaemia without ECMO [41]. They found that patients with higher doses and longer duration of vasopressor use had a higher likelihood of developing limb ischaemia. Patients who have an additional ECMO as a risk factor are even more likely to develop limb ischaemia [9]. In our study, the vast majority of patients (93.3%) required vasopressors on the day after ECMO implantation. There was no significant difference between the two groups (*p* = 0.668). The number of patients requiring this therapy did, however, decrease as time progressed. Although on day one after ECMO implantation, 93.3% of the patients required treatment with vasopressors, that percentage decreased to 76.9% on day three. This is due to the fact that, on one hand, patients recovered and required less circulatory support. On the other hand, some patients died within the first few days. Even though there was no significant difference between patients with and without limb ischaemia, the use of vasopressors should be not underestimated. Patients presenting with limb ischaemia should always be evaluated if vasopressors are possibly reducible.

The impact of cannula diameter on short-, mid- and long-term outcomes in patients placed on ECMO remains unclear [42,43]. Kim et al. found that patients with lower arterial cannula size (14–15 French (Fr.)) suffered significantly less from limb ischaemia compared to patients with a larger arterial cannula diameter (16–17 Fr.) [42]. Moreover, the ECMO duration period was significantly shorter in patients with smaller cannulas [42]. In addition, Takayama et al. mentioned that the use of big arterial cannulas (>17 Fr.) is associated with adverse outcomes in patients during ECMO support [43]. Furthermore, the authors found a significantly higher bleeding rate in the above-mentioned patient group. This all leads to the hypothesis that patients could benefit from smaller arterial cannulas [14,42,43]. The average diameter of arterial cannulas (*p* = 0.365) and distal perfusion cannulas (*p* = 0.976) did not differ significantly between both groups in our study.

In our study, more patients with additional IABP and Impella support were in the no limb ischaemia group. Generally, limb complications are more frequent in patients with an additional IABP support [39]. In contrast, limb ischaemia was reported less in patients with concomitant Impella implantation, with an incidence of 4–17% [40,41]. This is also most likely due to the smaller diameter of the cannula [42]. In addition, Impella is placed with the use of fluoroscopy in the catheter laboratory or open surgically [43]. Despite the high complication rate, already-mentioned strategies of left ventricular unloading could improve the outcome in ECMO patients [42,43].

Heparinisation of patients on ECMO is obligatory for the prevention of thromboembolic complications [44]. On the other hand, bleeding events also have a high incidence with heparinisation [45]. Therefore, Raman et al. compared different heparinisation strategies to find the best balance. They compared standard heparinisation (5.000 U of heparin with subsequent heparinisation with an activated clotting time between 180 and 220 s) with low heparinisation (5.000 U heparin without following heparin administration) [45]. They found that the low heparin strategy was not associated with adverse outcomes [45]. Therefore, a low heparin strategy could decrease the bleeding rate and might not impact the thromboembolic rate [44,45].

Thus far, there are few studies identifying relevant diagnostic markers of critical limb ischaemia [8,46]. Authors described peripheral arterial disease, diabetes and concomitant IABP therapy as predictors for acute limb ischaemia [8]. Therefore, patients with the above-mentioned risk factors must be monitored even more carefully to avoid potentially reversible complications [47,48]. In contrast, we could not identify any statistical differences regarding peripheral arterial disease (*p* = 0.458), diabetes (*p* = 0.713) and additive use of IABP (*p* = 0.589) in our analysis. However, larger prospective trials are necessary to identify the previously described risk factors of acute limb ischaemia during ECMO initiation.

### Limitations

The study was conducted as a retrospective trial with a relatively small number of patients. Moreover, the sample size was not calculated. Furthermore, we focussed only on short-term outcomes. The mid- and long-term outcomes and quality of life measures were not part of our study. Finally, we had only limited access to patient’s data in electronic or written form.

## 5. Conclusions

Our analysis showed that acute limb ischaemia is not associated with increased in-hospital mortality during ECMO therapy. However, the bleeding rate was significantly higher in the limb ischaemia group. Therefore, early diagnosis and prevention of acute limb ischaemia may decrease haemorrhage complications in patients that have been placed on ECMO.

## Figures and Tables

**Table 1:** Demographic data and relevant comorbidities (n = 104).

	Limb Ischaemia (n = 19)	No Limb Ischaemia (n = 85)	*p*-Value
Age, years, mean ± SD	56 ± 13	53 ± 13	0.399
Female, n (%)	8 (42.1%)	26 (30.6%)	0.333
BMI, kg/m^2^, mean ± SD	29.8 ± 7.8	28.4 ± 6.1	0.543
Myocardial infarction, n (%)	7 (36.8%)	18 (21.7%)	0.235
Stroke, n (%)	2 (10.5%)	2 (2.4%)	0.154
COPD, n (%)	3 (15.8%)	8 (9.5%)	0.421
Smoking, n (%)	8 (42.1%)	27 (32.1%)	0.408
CKD, n (%)	4 (21.1%)	7 (8.3%)	0.116
Dialysis, n (%)	1 (5.3%)	6 (7.1%)	0.769
Diabetes, n (%)	6 (31.6%)	23 (27.4%)	0.713
AH, n (%)	6 (31.6%)	40 (47.6%)	0.204
PAD, n (%)	1 (5.3%)	2 (2.4%)	0.458
Hyperlipidaemia, n (%)	6 (31.6%)	19 (22.6%)	0.393

BMI—body mass index; COPD—chronic obstructive pulmonary disease; CKD—chronic kidney disease; AH—arterial hypertension; PAD—peripheral arterial disease.

**Table 2:** ECMO-initiation data (n = 104).

	Limb Ischaemia (n = 19)	No-Limb Ischaemia (n = 85)	*p*-Value
CPR before ECMO, n (%)	13 (68.4%)	45 (52.9%)	0.219
Duration CPR, median (IQR)	22.50 (20.00–60.00)	9.00 (1.00–45.00)	0.819
Distance to patient (km), median (IQR)	35 (8–44)	38 (8–44)	0.546
Catecholamine before ECMO, n (%)	6 (85.7%)	30 (76.9%)	0.604
Implantation technique, PP, n (%)	16 (94.1%)	64 (92.8%)	0.843
Arterial canula (Fr.), median (IQR)	17.00 (17.00–19.00)	17.00 (17.00–18.00)	0.365
Venous canula (Fr.), median (IQR)	23.00 (21.00–23.00)	23.00 (21.00–23.00)	0.230
DPC canula, n (%)	12 (70.6%)	47 (71.2%)	0.960
DPC canula (Fr.), median (IQR)	6.50 (6.50–8.00)	7.00 (6.50–8.00)	0.976
Initial ECMO flow, L/m, median (IQR)	4.80 (3.00–4.00)	4.00 (2.50–2.50)	0.454
ECMO duration, median (IQR)	82.00 (46.00–144.00)	124.00 (45.50–203.00)	0.243
IABP, n (%)	0 (0.0%)	6 (7.1%)	0.589
Impella, n (%)	3 (15.8%)	7 (8.2%)	0.385
Vasopressor therapy day one after ECMO implantation, n (%)	18 (94.7%)	79 (93.0%)	0.668
Vasopressor therapy day two after ECMO implantation, n (%)	17 (89.5%)	73 (85.9%)	0.663
Vasopressor therapy day three after ECMO implantation, n (%)	13 (68.4%)	67 (78.8%)	0.706

ECMO—extracorporeal membrane oxygenation; IABP—intra-aortic balloon pump; Impella—circulatory support device; CPR—cardiopulmonary resuscitation; PP—per punctionem; DPC—distal perfusion cannula; Fr.—French.

**Table 3:** Laboratory data (n = 104).

	Limb Ischaemia (n = 19)	No Limb Ischaemia (n = 85)	*p*-Value
**Before ECMO**			
CK, U/L, median (IQR)	728.00 (404.00–4617.00)	782.00 (216.00–3693.00)	0.982
CK-MB, U/L, median (IQR)	131.50 (116.50–433.50)	178 (41.00–350.00)	0.995
Lactate (mmol/L), median (IQR)	9.36 (6.65–12.50)	7.76 (3.10–12.00)	0.986
Creatinine (mg/dL), mean ± SD	2.00 ± 0.90	2.40 ± 3.20	0.578
**24 h after ECMO**			
CK, U/L, median (IQR)	389.50 (404.00–8368.00)	1817.50 (232.00–3859.50)	0.251
CK-MB, U/L, median (IQR)	19.00 (14.00–179.00)	140.50 (28.00–302.00)	0.447
Lactate (mmol/L), median (IQR)	3.28 (1.58–8.80)	2.40 (1.74–7.11)	0.922
Creatinine (mg/dL), mean ± SD	2.20 ± 1.00	2.00 ± 1.40	0.607
**48 h after ECMO**			
CK, U/L, median (IQR)	246.50 (253.00–4948.00)	2396.00 (220.00–2567.00)	0.353
CK-MB, U/L, median (IQR)	12.50 (39.00–259.00)	91.50 (21.00–123.00)	0.211
Lactate (mmol/L), mean ± SD	3.90 ± 4.30	2.70 ± 2.30	0.165
Creatinine (mg/dL), mean ± SD	1.80 ± 0.90	1.80 ± 1.10	0.915
**72 h after ECMO**			
CK, U/L, median (IQR)	2281.00 (285.00–4530.00)	920.50 (202.00–1260.00)	0.148
CK-MB, U/L, mean ± SD	66.00 ± 62.00	48.00 ± 70.00	0.440
Lactate (mmol/L), mean ± SD	2.40 ± 1.80	1.80 ± 1.10	0.150
Creatinine (mg/dL), (IQR)	2.67 (1.11–2.08)	1.37 (0.98–2.16)	0.471
**96 h after ECMO**			
CK, U/L, median (IQR)	1378.50 (226.00–2489.00)	427.00 (98.00–910.00)	0.002
CK-MB, U/L, median (IQR)	27.50 (15.00–33.00)	19.00 (14.00–37.00)	0.002
Lactate (mmol/L), median (IQR)	1.44 (0.90–1.47)	1.19 (0.88–1.94)	0.693
Creatinine (mg/dL), median (IQR)	2.30 (1.29–2.28)	1.12 (1.00–2.00)	0.640

ECMO—extracorporeal membrane oxygenation; CK—creatine kinase; CK-MB—creatine kinase MB.

**Table 4:** Primary and secondary endpoints (n = 104).

	Limb Ischaemia (n = 19)	No Limb Ischaemia (n = 85)	*p*-Value
Surgical embolectomy, n (%)	10 (52.6%)	0 (0.0%)	<0.001
Surgical fasciotomy, n (%)	3 (15.7%)	0 (0.0%)	0.005
Stroke, n (%)	3 (15.8%)	8 (9.5%)	0.421
TE, n (%)	6 (31.6%)	18 (21.2%)	0.371
Bleeding, n (%)	14 (73.7%)	31 (38.3%)	0.005
Surgery due to bleeding, n (%)	3 (15.7%)	0 (0.0%)	0.005
ARDS, n (%)	3 (15.8%)	23 (27.7%)	0.387
HF, n (%)	7 (36.8%)	26 (30.6%)	0.596
GB, n (%)	1 (5.3%)	4 (4.8%)	0.927
RF, n (%)	13 (68.4%)	48 (56.5%)	0.339
Dialysis, n (%)	9 (47.4%)	30 (35.7%)	0.344
Wound infection, n (%)	1 (5.3%)	6 (7.1%)	0.769
SIRS, n (%)	8 (42.1%)	25 (29.8%)	0.298
Septic shock, n (%)	6 (31.6%)	28 (33.3%)	0.883
PPI, n (%)	1 (5.3%)	3 (3.5%)	0.560
ICU-duration days, median (IQR)	5 (2–23)	9 (4–13)	0.157
Mortality, n (%)	12 (63.2%)	51 (60.0%)	0.799

ICU—intensive care unit; ARDS—acute respiratory distress syndrome; RF—renal failure; HF—hepatic failure; GB—gastrointestinal bleeding; TE—thromboembolic events; PPI—permanent pacemaker implantation; SIRS—systemic inflammatory response syndrome.

## Data Availability

Data are available on a special request.
